# CD28null and Regulatory T Cells Are Substantially Disrupted in Patients with End-Stage Renal Disease Due to Diabetes Mellitus

**DOI:** 10.3390/ijms22062975

**Published:** 2021-03-15

**Authors:** Erasmia Sampani, Dimitra-Vasilia Daikidou, George Lioulios, Aliki Xochelli, Zoi Mitsoglou, Vasiliki Nikolaidou, Chrysostomos Dimitriadis, Asimina Fylaktou, Aikaterini Papagianni, Maria Stangou

**Affiliations:** 1 Department of Nephrology, Aristotle University of Thessaloniki, Hippokration Hospital, 56442 Thessaloniki, Greece; sampaniedoc@gmail.com (E.S.); dvdaikidou@gmail.com (D.-V.D.); pter43@yahoo.gr (G.L.); zoimhts@gmail.com (Z.M.); chrysdim@gmail.com (C.D.); aikpapag@otenet.gr (A.P.); 2 National Peripheral Histocompatibility Center, Department of Immunology, Hippokration Hospital, 56442 Thessaloniki, Greece; aliki.xochelli@gmail.com (A.X.); basoniko@hotmail.com (V.N.); fylaktoumina@gmail.com (A.F.)

**Keywords:** diabetes mellitus, end-stage renal disease, lymphocytes, CD28null cells, regulatory T cells

## Abstract

Background: End-stage renal disease (ESRD) is associated with alterations in T-cell immunity, including increased CD28null and reduced regulatory T cells (Tregs). However, whether immune disturbances are due to ESRD or primary disease is not yet clear. As diabetes mellitus is the leading cause of ESRD, we evaluated its impact on the immune profile of ESRD patients. Methods: CD28null, Tregs, and natural killer cells were initially analyzed by flow cytometry in 30 predialysis ESRD patients due to diabetes (DM), 30 non-DM (NDM), and 25 healthy controls. Measurements were repeated after 6 months on hemodialysis (HD) or peritoneal dialysis (CAPD). Results: The percentage of CD4 + CD28null cells, CD8 + CD28null cells, and Tregs showed significant differences in DM, NDM, and controls; mean rank 33.71 vs. 25.68 vs. 18.88, *p* = 0.006, 37.79 vs. 28.82 vs. 17.08, *p* = 0.008, and 20.79 vs. 26.12 vs. 41.33, *p* = 0.001, respectively. DM vs. NDM had increased CD4 + CD28null and CD8 + CD28null cells, 11.5% (1.5%–24%) vs. 4.1% (0–42.3%), *p* = 0.02 and 61.3% (24%–76%) vs. 43% (5.7%–85%), *p* = 0.04, respectively. After 6 months on HD but not CAPD, DM showed a significant further increase in CD4 + CD28null cells, from 30 (14–100) to 52.7 (15–203), *p* = 0.02; and CD8 + CD28null cells, from 137 (56–275) to 266 (103–456), *p* = 0.01. Conclusions: Diabetes mellitus affects T-cell subtypes even at predialysis stage, though changes become more prominent after commencement on HD.

## 1. Introduction

Chronic metabolic acidosis, oxidative stress, the accumulation of uremic toxins, and low-grade inflammation are some of the consequences of end-stage renal disease (ESRD) [1,2,3]. They directly affect almost every system, including the immune system, which is the overarching surveillance mechanism to control almost each function [4,5]. Immunological complications in chronic kidney disease encompass the exceptional characteristic of combined immune deficiency happening together with chronic inflammation [4,6,7].

Immune disorders and clinical consequences gradually progress during the deterioration of renal function towards reaching ESRD, and they are characterized by an increased risk of cardiovascular disease, infections, and malignancies, leading to increased morbidity and mortality [8,9,10]. Changes in white-cell count and their subtypes are evident, but alterations in T-cell subpopulations seem to play a prominent role in the development of long-term clinical complications [11,12]. Three different molecules on T cells are primarily affected, namely, CD28, FoxP3, and CD25 in ESRD patients, leading to an increase in CD4 + CD28null and CD8 + CD28null cells, and a reduction in regulatory T (Treg) cells [11,12,13,14,15]. The activation of naive T cells requires antigen recognition, but also an interaction of B7 with the CD28 receptor. CD28 in the only costimulatory receptor constitutively expressed on naive T cells; therefore, its presence is fundamental for the initiation and maintenance of T-cell-mediated immune responses [16,17]. In chronic-inflammation situations such as chronic kidney disease and diabetes mellitus (DM), the transformation of receptor repertoire on T cells, including the loss of the CD28 molecule, results in a cell type of which the phenotype and activities resemble NK cells, called the NK-like T cell [2,10,11,18,19].

Treg cells are characterized as the firefighters of the immune system [20]. Activated Treg cells produce TGF-β and IL-10, but also reduce the production of IL-2, TNF-α, and IL-5; thus, they suppress the differentiation of Th1 and Th2 cells, and reduce expression of Major Histocompatibility Complex (MHC) and their costimulatory molecules on antigen-presenting cells (APCs). Therefore, Treg cells are responsible for the downmodulation of immune responses, the maintenance of peripheral tolerance, and the prevention of autoimmune diseases [20,21].

Chronic kidney disease may not itself be the only reason for immune changes; the primary disease that caused the kidney damage also participates, contributes, and sometimes modifies the process of immune deficiencies. Diabetes mellitus (DM) is a systemic disorder with serious detrimental effects to almost all organs, leading to tissue damage, organ failure, and is nowadays the leading cause of ESRD [22]. Hemodynamic mechanisms and metabolic factors in DM are responsible for the activation of proinflammatory signaling pathways, and also T- and B-cell immunity [12,18,23].

With the above-mentioned stimulation of immune mechanisms in patients with ESRD, and the influence of DM on the immune profile, it would be interesting to evaluate immune deficiencies in patients with ESRD due to diabetic nephropathy. In the present study, we investigated whether DM acts as an additional parameter in specific immunological disorders seen in ESRD patients.

## 2. Results

Sixty ESRD patients, 30 DM and 30 NDM, were initially included; 4 (2 DM and 2 NDM) subsequently withdrew, 3 were lost to follow-up, and 1 died. Twenty-five healthy volunteers, of similar age and the same sex, comprised the control group.

Table 1 and Table 2 describe the clinical and laboratory features of the two groups of patients at ERSD-T0.

### 2.1. Alterations of T Lymphocytes and Their Subpopulations Patients at ESRD-T0

#### 2.1.1. Differences in Neutrophils and Lymphocyte Cells

The whole cohort of patients at predialysis ESRD (ESRD-T0) had increased WCC numbers compared to those of the controls: 7457 ± 2264/μL vs. 6726 ± 1314/μL, *p* = 0.04, increased neutrophil, total cell count, 5259 ± 1942/μL vs. 4061 ± 1444/μL, respectively, *p* = 0.01, and percentage 68.9 ± 8.3 vs. 59.6 ± 13.2, respectively, *p* = 0.003, and reduced lymphocyte count 1371 ± 589/μL vs. 2198 ± 556/μL, respectively, *p* < 0.0001 and percentage of lymphocytes 18.8 ± 6.5 vs. 33.6 ± 9.4, respectively, *p* < 0.0001.

**Figure 1:**
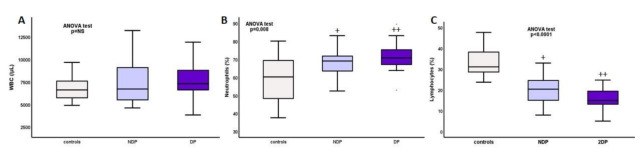
Total numbers of (**A**) white cells, (**B**) neutrophils (%), and (**C**) lymphocytes (%) in DM, NDM, and controls. + *p* NDM vs. controls <0.01, ++ *p* DM vs. controls <0.0001.

Differences between DM, NDM, and controls are depicted on Table 2. Total white-cell count was increased in DM and NDM patients at ESRD-T0 compared to controls, and this difference was probably due to neutrophils, as both their percentage and total count were increased, while lymphocytes were significantly reduced, with DM having the highest levels of neutrophils and lowest lymphocytes, percentage, and total counts (Figure 1).

Moreover, the ratio of neutrophil/lymphocyte count (NLR) was significantly increased in DM and NDM compared to that in the controls, with mean rank 37.85 vs. 31.57 vs. 12.73, respectively, *p* < 0.0001; and the ratio of neutrophil/lymphocyte percentage, mean rank 37.65 vs. 31.67 vs. 12.67, respectively, *p* < 0.0001.

#### 2.1.2. Differences in CD3+CD4+ and CD3+CD8+ Cells

**Figure 2:**
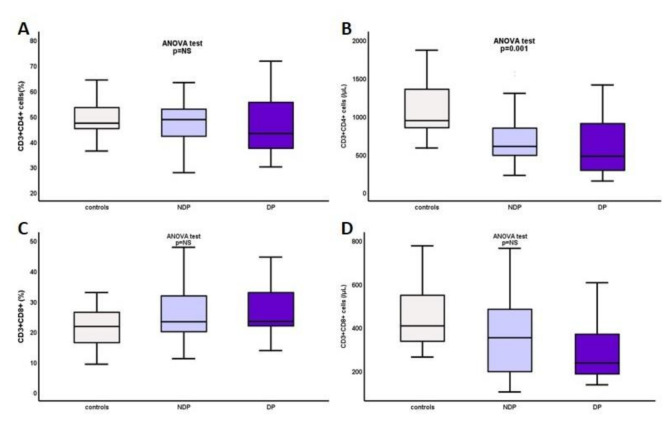
(**A**, **B**) CD3+CD4+ percentage and total count, and (**C**, **D**) CD3+CD8+ percentage and total count in DM, NDM, and controls.

ESRD-T0 patients had significantly reduced numbers of CD3+CD4+ cells, 672 ± 357/μL vs. controls, 1100 ± 375/μL, *p* < 0.0001, with no significant difference in CD3+CD8+ population. Comparing DM, NDM, and controls, CD3+CD4+ cells was significantly reduced in DM and NDM, 622.67 ± 408.7/μL vs. 693.89 ± 338.5/μL vs. 1100.14 ± 375.4/μL, respectively, *p* = 0.001, while there were no significant differences regarding CD3+CD8+ cells (Figure 2).

The ratio of CD3+CD4+/CD3+CD8+ lymphocytes was reduced in DM and NDM compared to that in the controls, but differences could not reach statistical significance, Mean Rank 26.58 vs. 29.82 vs. 33.33, respectively, *p* = NS. Again, the lowest levels were seen in DM, however with no significant difference between DM and NDM.

#### 2.1.3. Increase in CD4+CD28null and CD8+CD28null Cells in ESRD and DM

CD28null cells were significantly increased in the whole cohort of ESRD-T0 patients compared to that in the controls, 20.46% (1%–50.1%) vs. 7.2% (1%–17.2%), *p* = 0.005, and total number 173 (13–1209)/μL vs. 154 (26–430)/μL, *p* = NS, respectively. Similar differences were seen in CD4+CD28null cells, 7.5% (0–43%) vs. 2.6% (0.1%–7.8%), *p* = 0.03, and total number 25.7 (0–550)/μL vs. 32 (0.9–76)/μL, *p* = NS, respectively; and CD8+CD28null cells, 55% (5.7%–85%) vs. 39% (7.8%–57%), *p* = 0.02, and total count 129(13–735)/μL vs. 116(25–391)/μL, *p* = NS, respectively.

**Figure 3:**
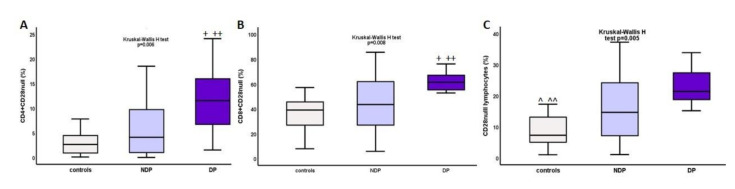
Differences in percentage of (**A**) CD4+CD28null cells, (**B**) CD8+CD28null cells, and (**C**) total CD28null cells between DM, NDM, and controls. * *p* DM vs. controls <0.0001, ** *p* DM vs. NDM <0.04, ^ *p* NDM vs. controls 0.04, ^^ *p* DM vs. controls <0.0001.

Comparing DM, NDM, and controls, the percentage of all CD28null cells was increased, mean rank of 32.54 vs. 27.42 vs. 25.15, *p* = 0.005, as was the percentage of CD4+CD28null cells, mean rank of 33.71 vs. 25.68 vs. 18.88, *p* = 0.006, and the percentage of CD8+CD28null cells, 37.79% vs. 28.82% vs. 17.08%, *p* = 0.008, respectively. The above differences are depicted on Figure 3. Significant difference was also seen between DM and NDM in terms of the percentage of CD4+CD28null cells, mean rank 28.25 vs. 18.8, *p* = 0.02 and CD8+CD28null cells, mean rank 28.5 vs. 19.75, *p* = 0.04.

#### 2.1.4. Changes in Natural Killer Cells and Tregs

**Figure 4:**
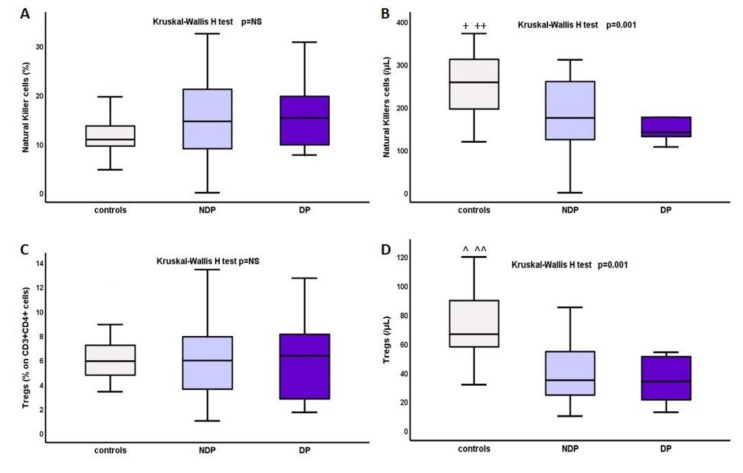
Differences in percentage and total cell count in (**A**,**B**) NK cells and (**C**,**D**) Tregs in DM, NDM, and controls, * *p* (DM vs. controls) = 0.03, ** *p* (DM vs. controls) = 0.001, ^ *p* (DM vs. controls) = 0.02, ^^ *p* (DM vs. controls) = 0.001.

Total numbers of NK cells and Tregs were significantly reduced in the whole cohort of ESRD-T0 patients compared to that in the controls, median range: 258.13(119–1060) vs. 168.15(0–617.5)/μL, mean rank of 26.82 vs. 39.33, *p* = 0.01 respectively, for NK cells; and 65.2(30–114.7) vs. 29.19(3.1–113.7), 24.29 vs. 42.2, *p* < 0.0001, respectively, for Tregs. Kruskal–Wallis H test also showed significant differences between DM, NDM, and controls for both NK cells and Tregs, as depicted in Table 2 and Figure 4, although there was no significant difference between DM and NDM.

### 2.2. Influence of Dialysis on T Lymphocytes—Different Effects after Applying HD or CAPD

Supplementary Tables S1 and S2 depict changes in the immunological profile of NDM and DM patients after being on hemodialysis (HD) or continuous ambulatory peritoneal dialysis (CAPD) for 6 months. A reduction in WCC and neutrophils was noticed in both groups, while total lymphocyte count was significantly increased mainly due to the increase in CD3+CD4+ cells. A reduction in CD28null cells, and both subtypes, CD4+CD28null and CD8+CD28null cells, was evident in NDM but not in DM, in whom both these T-cell subpopulations were increased after the initiation of dialysis. NK cells were also increased in DM, though not significantly, and remained stable in NDM, while Treg cells remained mostly the same in DM and increased in NDM.

In subgroup analysis, DM undergoing HD for 6 months showed a significant increase in CD3+CD8+ and NK cells, and both CD4+CD28null and CD8+CD28null. Instead, DM who had commenced on CAPD showed a reduction in all the above cell types. Similar, but not so prominent, differences between HD and CAPD methods were seen in NDM after a 6 month follow up (Supplementary Table S2).

## 3. Discussion

In the present study, we evaluated the effect of ESRD and DM on T-cell immunity. We initially analyzed T-cell subpopulations, including CD4+, CD8+, CD4+CD28null, CD8+CD28null, and regulatory T and natural killer cells in diabetic and nondiabetic patients on ESRD. The study was conducted at two phases. During the first, all measurements were performed in patients at predialysis ESRD; in the second, we evaluated patient outcome after being on dialysis for a period of 6 months. In the second phase, patients were divided into subgroups according to the applied dialysis method, either HD or CAPD, and the effects of the two methods on T-cell immunity were assessed in DM and NDM.

Patients with predialysis ESRD showed differences in white-cell count with a significant increase in neutrophils and a reduction in lymphocyte count, leading to an increase in neutrophil/lymphocyte ratio. These results agree with previous studies that described relative lymphopenia affecting primarily naïve CD4+ cells and, to a lesser degree, naïve CD8+ cells, attributed to impaired circulating levels of the IL-7 cytokine in chronic kidney disease [24]. The balance and ratio of certain lymphocyte subpopulations was disrupted in ESRD-T0 patients, with a reduction in CD4+ cells leading to reduced CD4/CD8 ratio, a reduction in NK and Treg cells, and an increase in CD4+CD28null and CD8+CD28null cells compared to those in the controls.

The presence of DM had a significant impact only on the elimination of CD28 receptor on T-cell surfaces, leading to an increased frequency of both CD4+CD28null and CD8+CD28null cells. 

Multiple pathways that lead to tissue damage are activated in DM, initializing by hyperglycemia and the increased production of advanced glycation end products (AGEs) and their receptors, leading to polyol and hexosamine pathway activation, and protein kinase C (PKC) isoform production. Nonetheless, the immune system seems to be seriously affected, with oxidative and endoplasmic reticulum stress being major participants in the chronic inflammatory reactions of DM [24,25,26]. Both innate and adaptive immunity are affected; the diabetic milieu causes the activation of multiple signaling cascades, such as Janus kinase/signal transducer and activator of transcription (JAK-STAT), p38 mitogen-activated protein kinase (p38-MAPK) and TNF pathways, the increased expression of adhesion molecules, intracellular and extracellular receptors, and the production of proinflammatory cytokines (IL-1, IL-6, TNF-α, TGF-β), resulting in low-grade sustained inflammation [18,24,27,28,29]. The influence of adaptive immunity has not been extensively studied in DM, although the activation of T and B cells has recently attracted interest [28,29,30]. Thus, the effect of IL17 on the progression of inflammation and proteinuria has been proven in experimental diabetic nephropathy, while regulatory T cells seem to have opposing effects by suppressing autoimmunity and ameliorating immune responses [31,32]. Moreover, human studies showed the activation of T cells in diabetic patients, but also synergistic activity between T lymphocytes and the TNF-α signaling pathway in the development and progression of DM [30].

In the present study, the main alterations in T cells due to DM implicated CD28null cells, affecting both CD4+ and CD8+ compartments. These results agree with previous studies that described increased CD4+CD28null cells even in a pediatric population, correlating with the development of microalbuminuria the presence of neurologic complications [8], microvascular changes, and the progression of atherosclerotic disease [33].

Chronic kidney disease acts in a similar way, as a cause of T-cell abnormalities, with a shift to the immunosenescent phenotype, including a reduction in CD45RA+CD28+ naïve subsets and an increase in CD28null cells [11,12,34]. On the basis of our results, we cannot definitely conclude whether DM or ESRD plays the most significant role in the T-cell abnormalities seen in our population. Checking the above T-cell subtypes in diabetic patients without renal impairment could be helpful; nevertheless, the two diseases could synergistically contribute to the development of an immunosenescent and immunocompromised phenotype.

Moreover, prospective subgroup analysis following patients’ commencement on dialysis further supported the initial findings and established the role of DM in specific immunological changes. We evaluated the impact of HD and CAPD in further alterations of the T-cell profile of DM and NDM. The general impression was a beneficial effect of dialysis in both groups, including a reduction in total white-cell and neutrophil count, and a significant increase in lymphocytes, mainly CD4+ cells. However, an unfavorable effect of dialysis was also observed, but restricted to DM only, regarding CD28null cells and NKs, which were elevated after 6 months on dialysis. In subgroup analysis, DM who had started on HD had a significant further increase in CD8+, CD4+CD28null, CD8+CD28null, and NK cells. Instead, those DM who had started on CAPD had a reduction in CD28null cells, especially CD4+CD28null cells simultaneously with an increase in Treg cells. In NDM, results were similar, but the effect of HD on CD28 expression was not that prominent, and there was a beneficial effect of CAPD on CD4+CD28null, CD8+CD28null, and Treg cells. The different ways in which dialysis methods affect T lymphocyte subpopulations and their strong impact on mainly diabetic patients is a completely novel finding. Although the effect of ESRD on the expression of the CD28 molecule was proven, and many investigators agree that patients on chronic kidney disease stages IV–V show a significant increase in the frequency of CD4+CD28null cells, the further effect of dialysis is controversial, with some investigators finding no differences between predialysis and dialysis-dependent ESRD patients, and others showing CD28null cells further expanding in patients on HD [10,35,36]. However, all previous studies focused only on CD4+CD28null cells, excluding CD8+CD28null cells as clinically unimportant. Only recently were CD8+CD28null cells found to accumulate in atherosclerotic plaques and act as independent predictors of cardiovascular events in ESRD. Regardless of the presence of DM, HD and CAPD have opposing effects in the existence and accumulation of CD28null cells, with CAPD causing a significant amelioration in CD28null cells [10]. In the present study, patients with ESRD due to diabetic nephropathy had a further significant increase in these cells, while nondiabetics were not severely affected by HD. Our findings suggest that patients with ESRD due to DM should be very carefully considered for CAPD, especially if they have a cardiovascular disease since the method has a definite beneficial effect on CD28null cells.

However, there are some limitations in our study. A group of DM patients without diabetic nephropathy could support the results and further elucidate the impact of DM on CD28null and Treg cells. The strength of our study is based on the prospective evaluation of T-cell subpopulations, and the different ways in which the two groups of patients, DM and NDM, behaved. This is the first time that it was proven that we can affect the immunological profile of ESRD patients by applying different dialysis methods.

On the basis of our results, DM seems to act as an additive factor to exacerbate the detrimental effects of chronic kidney disease in predialysis patients, but also stands as a bad prognostic factor in the outcome of these patients after commencing dialysis. In both groups, CAPD had a beneficial effect, while HD had an unfavorable effect on immunologic profile mainly by upregulating CD28 expression and increasing Treg cells, but this was more prominent in diabetic compared to in nondiabetic patients.

## 4. Materials and Methods

The present study investigated changes in T lymphocyte subpopulations in diabetic and nondiabetic patients with end-stage renal disease (ESRD) before commencing dialysis, and further effects of applied dialysis methods.

### 4.1. Study Schedule 

Patients with ESRD that had not started on dialysis were included in this prospective longitudinal study. Day of enrollment (ESRD-T0) was the day on which they had commenced on a dialysis method, either hemodialysis (HD) or continuous ambulatory peritoneal dialysis (CAPD). Patients were monitored for 6 months. All patients were eligible for both methods; the choice of the method was based only on individual preference and socioeconomic status. The study population was divided into two groups, diabetic (DM) and nondiabetic (NDM) patients. Patients were on regular follow-up with clinical and laboratory investigation every month until the end of follow-up (ESRD-T6).

The study was conducted in two phases: during the initial phase, T lymphocyte subpopulations, including CD4, CD8 cells, their expression of the CD28 antigen, and T regulatory (Tregs) and natural killer (NK) cells, were estimated in all patients at ESRD-T0. In the second phase, the effect of dialysis was evaluated in both groups, DM and NDM patients after 6 months. We eventually performed subgroup analysis estimating T-cell alterations in four subgroups, NDM who had commenced HD or CAPD (Subgroups 1 and 2, respectively) and DM who had started on HD or CAPD (Subgroups 3 and 4, respectively).

### 4.2. Patients

Patients in the study had to: be 18–75 years old; have been under regular investigation for the last 12 months; and have adequate control of anemia, diabetes mellitus, dyslipidemia, hypertension, hyperparathyroidism, and gradual progression to ESRD. Patients with a recent infection (<6 months), history of malignancy, systemic autoimmune disease, or treatment with corticosteroids or immunosuppressive agents during the last 12 months prior to enrollment were excluded. After enrollment, patients had to be stable on the dialysis method that they chose in the beginning of the study. Patients who changed method, could not reach adequate dialysis levels, or had an infection episode were withdrawn from the study.

For both groups, demographic and clinical features, medical history, and medication were recorded at ESRD-T0. Biochemical, hematological, and immunological profiles, including an assessment of T-cell subpopulations were established and evaluated at ESRD-T0 and ESRD-T6. Blood samples at ESRD-T0 were drown just before starting on HD or CAPD, while the follow up samples, at ESRD-T6, were collected at the beginning of a midweek HD session.

During follow-up, HD patients had to be stable, on a regular hemodialysis timetable of three sessions per week for at least 4 h per session. Dialysis membranes were biocompatible, and patients were dialyzed through arteriovenous fistula (AVF) performed at least 1 month prior to first HD session. Similarly, CAPD patients had to have their catheter insertion at least 1 month before commencing on the method, with no surgical complications and no episodes of peritonitis or catheter infections from the day of insertion until the end of follow up.

The adequacy of dialysis was assessed by KT/V calculation, which was performed twice during the study, at Month 3 and at the end of follow up, Month 6 (ESRD-T6). At the end of 3 months, HD or CAPD patients who, did not reach KT/V > 1.2 per session or KT/V > 1.7 per week, respectively, were withdrawn from the study.

Twenty-five healthy nondiabetic volunteers, matched for age, sex, and race served as controls.

The study protocol followed the general principles of the Declaration of Helsinki (2008 Amendment) and was approved by the Ethics Committee of Hippokration Hospital of Thessaloniki. All subjects provided informed consent prior to study enrollment.

### 4.3. Laboratory Measurements

#### Flow Cytometry

**Figure 5:**
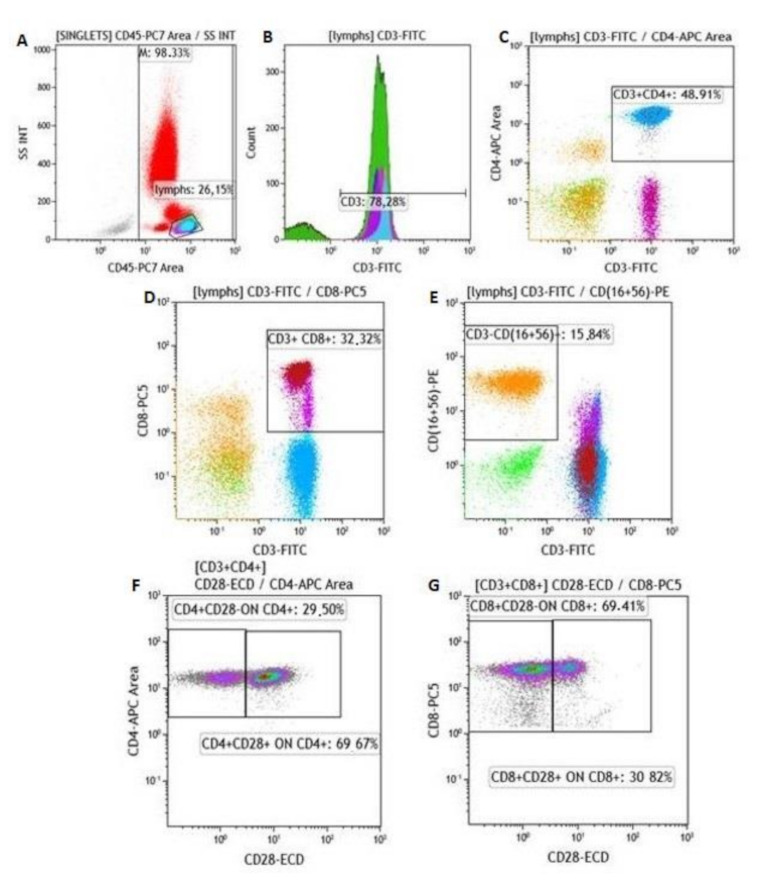
Description of gating process. (**A**) Gating of lymphocytes and identification of (**B**) CD3+, (**C**) CD3+CD4+, (**D**) CD3+CD8+, (**E**) CD3–CD(16+56)+ cells, CD28– cells on CD4+ population, (**F**) CD4+CD28null cells and CD28– cells in CD8+ population, (**G**) CD8+CD28null cells.

**Figure 6:**
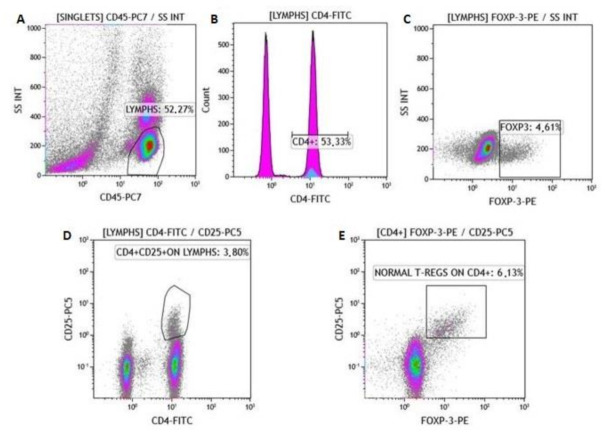
FOXP-3 assay—description of gating process. (**A**) Gating of lymphocytes. Identification of (**B**) CD4+, (**C**) FOXP-3 (+), and (**D**) CD4+CD25+ cells; (**E**) identification of normal Tregs on CD4+ cells.

Active protein C (APC) blood-collecting tubes were used for the collection of whole-blood samples from patients at ESRD-T0 and ESRD-T6, and controls. Blood samples were processed for total lymphocyte count and subgroups. Lymphocyte populations were determined with a cell counter (Navios Flow Cytometer, Beckman Coulter). Lymphocyte subtypes were evaluated by using a panel of monoclonal antibodies (Beckman Coulter), specifically, CD45-PC7 for lymphocytes, CD4+APC for CD3+CD4+ cells, CD8-PC5.5 for CD3+CD8+ cells, CD3-FITC/(CD16+CD56+)-PE for NK cells, CD25-PC5, Fox P3-PE for Tregs, CD28-ECD for CD4+CD28+, CD4+CD28–, CD8+CD28+ and CD8+CD28– cells. Figure 5 and Figure 6 show the gating process and procedure for singlet isolation.

Absolute numbers and percentages of lymphocytes (CD45+ cells), CD3+CD4+, CD3+CD8+, NK cells, Tregs, CD4+CD28+, CD4+CD28null, CD8+CD28+, and CD8+CD28null cells were estimated.

In all patients, the immunological risk profile (IRP) was estimated as was described before on the basis of the levels of CD4+ and CD8+ cells, the CD4/CD8 ratio, and the percentage of CD28null cells [10].

## 5. Statistics

Statistical analysis was performed with the Statistical Package for Social Sciences (SPSS Inc., Chicago, IL, USA) for Windows, version 25.0. Values of *p* < 0.05 (two-tailed) were considered to be statistically significant for all comparisons. Normally distributed continuous variables, as characterized by the Shapiro–Wilk and/or Kolmogorov–Smirnov test(s) were expressed as mean ± standard deviation, while data from nonparametric variables were expressed as medians and range. Differences between groups were estimated by Student’s t-test for paired and independent samples; an ANOVA test for normally distributed variables; and Mann–Whitney U test, Wilcoxon signed-ranks test, and Kruskal–Wallis H test for nonparametric variables. Subgroup analysis was performed on DM and NDM patients who started in HD or CAPD. Changes in T-cell subsets in each subgroup during dialysis were estimated, and differences were compared by Mann–Whitney U test.

## Figures and Tables

**Table 1:** Clinical characteristics of diabetic (DM) and nondiabetic (NDM) patients at ESRD-T0.

	DM	NDM	*p*
**N**	**28**	**28**	
Systolic blood pressure (SBP)	144 ± 26	140 ± 25	NS
Diastolic dlood pressure (DBP)	85 ± 23	74 ± 31	NS
Intimal–medial thickness (IMT)	0.06 ± 0.01	0.05 ± 0.01	NS
IMTmax	0.16 ± 0.23	0.06 ± 0.01	NS
**Comorbid conditions or habits**			
Smoking (%)	14 (50)	13 (46.42)	NS
Coronary artery disease (%)	19 (67.85)	16 (57.14)	NS
Peripheral artery disease (%)	13 (46.42)	4 (14.28)	NS
Stroke (%)	2 (7.14)	1 (3.5)	NS
Dyslipidemia (%)	19 (67.85)	16 (57.14)	NS

**Table 2:** Lymphocytes and T-cell subsets in patients (DM, NDM) and controls; *p* value calculated for differences between three groups (ANOVA or Kruskal–Wallis H test), and between DM and NDM (Mann–Whitney U test). WCC, white-cell count; NLR, neutrophil/lymphocyte count; IRP, immunological risk profile.

	DM	NDM	Controls	*p*	*p* (DM vs. NDM)
**N**	28	28	25		
WCC (K/μL)	7600 ± 2076	7399 ± 2365	6726 ± 1314	NS	NS
Neutrophils (K/μL)	5927 ± 1729	5029 ± 1985	4061 ± 1444	0.02	NS
Neutrophils (%)	72 ± 10	68 ± 7.5	59 ± 13	0.008	NS
Lymphocytes (K/μL)	1212 ± 564	1435 ± 596	2198 ± 556	0.0001	NS
Lymphocytes (%)	16.5 ± 9.4	19.5 ± 6	33.6 ± 9.4	0.0001	NS
NLR	6 ± 4.9	3.9 ± 2	2 ± 1.1	0.0001	NS
**T-cell subtypes**					
CD3+CD4+ cells	622(150–1412)	693.9(224–1579)	943(584–1876)	0.001	NS
CD3+CD4+ cells (%)	43(30–71)	48.7(27–63)	49.6(36–66)	NS	NS
CD3+CD8+ cells	235(135–600)	352(102–149)	406(263–775)	NS	NS
CD3+CD8+ cells (%)	23(11–44)	23(11–47)	22(9–33)	NS	NS
CD4+/CD8+	1.6(0.7–4.8)	1.9(0.7–4.3)	2.3(1–7)	NS	NS
CD28null cells	206.8(61–500)	162.5(12.6–1209)	155(26–430)	NS	NS
CD28null (%)	21.3(4.4–34)	14.6(1–50)	7.2(1–17)	0.005	0.02
CD4+CD28null	34.7(14–279)	20.9(0–550)	32(0.9–76)	NS	NS
CD4+CD28null (%)	11.5(1.5–24)	4.1(0–42.3)	2.6(0.1–7.8)	0.006	0.02
CD8+CD28null	132(47–400)	114(12–735)	116(25–391)	NS	NS
CD8+CD28null (%)	61.3(24–76)	43(5.7–85)	39(7.8–57)	0.008	0.04
Natural killer cells	141(56–460)	174.6(0–617)	258(119–1060)	0.01	NS
Naural killer cells (%)	15.3(7.7–31)	14.6(0–32.5)	10.9(4.7–40)	NS	NS
Treg cells	33.7(12–102)	34.7(9.9–130)	66.3(31–119)	0.001	NS
Treg cells (%)	3.1(1.1–6.7)	2.8(0.62–8.5)	2.9(1.6–5.7)	NS	NS
IRP	3(1–4)	3(1–4)	2(0–4)	0.006	NS

## Data Availability

Data available on request due to privacy. The data presented in this study are available on request from the corresponding author. The data are not publicly available because they are part of the authors’, E.S., PhD, which has not been published yet.

## Supplementary Materials

The following are available online at https://www.mdpi.com/1422-0067/22/6/2975/s1. Supplementary Table S1. Changes in lymphocyte subpopulations after 6 months on dialysis—subgroup analysis for nondiabetic and diabetic patients started on HD or CAPD. Supplementary Table S2. Changes in lymphocyte subpopulations after 6 months on dialysis—subgroup analysis for nondiabetic and diabetic patients started on HD or CAPD.
