# Supplementary material for: CD28null and Regulatory T Cells Are Substantially Disrupted in Patients with End-Stage Renal Disease Due to Diabetes Mellitus
Source: Int J Mol Sci. 2021 Mar 15;22(6):2975. doi: 10.3390/ijms22062975 (PMC8001943; doi:10.3390/ijms22062975)
Supplement: Supplementary file 1 [file ijms-22-02975-s001.pdf]

Supplementary Materials

Supplumentary Table 1. Changes in lymphocyte subpopulations after 6 months on dialysis—subgroup analysis for nondiabetic and diabetic patients started on HD or CAPD.

Nondiabetic Patients (NDM)

	**All NDM**			**HD**			**CAPD**		
	**ESRD-T0**	**ESRD-T6**	**p**	**ESRD-T0**	**ESRD-T6**	**p**	**ESRD-T0**	**ESRD-T6**	**p**
WCC (K/μL)	7399 ± 2356	6614 ± 1687	0.05	8558 ± 2676	6786 ± 1867	0.007	6116 ± 1384	6400 ± 1483	NS
Neutrophils (K/μL)	5029± 1824	3928 ± 1166	0.01	5889 ± 2403	3885 ± 1571	0.08	4236 ± 1126	3881 ± 863	NS
Neutrophils (%)	68.6 ± 7.2	62.7 ± 10.2	0.02	71.8 ± 7.2	60.3 ± 10.3	0.08	66 ± 6.5	62.8 ± 10.6	NS
Lymphocytes (K/μL)	1435 ± 627	1648 ± 581	0.05	1581 ± 756	1735 ± 655	NS	1300 ± 392	1538 ± 477	0.04
Lymphocytes (%)	19.8 ± 5.8	25.2 ± 7.5	0.001	18.2 ± 5.4	25.7 ± 8.4	0.003	21.7 ± 6	24.6 ± 7	NS
CD3+CD4+ cells	693.9 ± 338	848.6 ± 365.6	0.03	799 ± 404	899 ± 404	NS	619 ± 232	785 ± 316	0.02
CD3+CD4+ cells (%)	47.9 ± 8.2	51 ± 8.2	NS	50 ± 5.9	51 ± 4.9	NS	47.8 ± 10.5	50.5 ± 11	NS
CD3+CD8+ cells	387 ± 240	420.5 ± 230	NS	394 ± 296	422 ± 263	NS	360 ± 155	418 ± 193	NS
CD3+CD8+ cells (%)	26 ± 8	24.6 ± 8.3	NS	23 ± 7.6	22 ± 7.1	NS	27.4 ± 8.3	27 ± 9.5	NS
CD3+CD4+/CD3+CD8+	2.1 ± 0.9	2.3 ± 0.9	NS	2.2 ± 0.9	2.4 ± 0.8	NS	2 ± 0.9	2.2 ± 1.1	NS

Diabetic Patients (DM)

	**All DM**			**HD**			**CAPD**		
	**ESRD-T0**	**ESRD-T6**	**p**	**ESRD-T0**	**ESRD-T6**	**p**	**ESRD-T0**	**ESRD-T6**	**p**
WCC (K/μL)	7600 ± 2076	7361 ± 2134	NS	7945 ± 2373	7333 ± 2274	NS	6822 ± 1029	7425 ± 2101	NS
Neutrophils (K/μL)	5927 ± 1729	4535 ± 1731	0.05	7179 ± 1692	5894 ± 1755	0.03	3959 ± 363	3727 ± 182	NS
Neutrophils (%)	71 ± 10	66 ± 3.4	NS	80.6 ± 10	69.7 ± 1.4	NS	64 ± 15.8	65.7 ± 4.9	NS
Lymphocytes (K/μL)	1213 ± 564	1646.2 ± 684	0.03	1015 ± 437	1617 ± 755	0.02	1657 ± 618	1711 ± 587	NS
Lymphocytes (%)	16.5 ± 7.4	22.2 ± 5	0.02	13.2 ± 4.9	22 ± 5.9	0.005	24 ± 6.9	22.8 ± 2	NS
CD3+CD4+ cells	622.7 ± 408	753.5 ± 320	0.05	505 ± 363	671 ± 304	NS	885 ± 427	938 ± 315	NS
CD3+CD4+ cells (%)	47.2 ± 13.6	47 ± 15.3	NS	45 ± 15	42.8 ± 14	NS	52.1 ± 9.6	56.4 ± 15	NS
CD3+CD8+ cells	300 ± 147.9	467 ± 416	NS	266 ± 130	516 ± 473	0.07	377 ± 176	356 ± 271	NS
CD3+CD8+ cells (%)	26.2 ± 9	26.2 ± 12.4	NS	27.8 ± 10	29.2 ± 13	NS	22.7 ± 5.7	19.4 ± 8	NS

Supplumentary Table 2. Changes in lymphocyte subpopulations after 6 months on dialysis—subgroup analysis for nondiabetic and diabetic patients started on HD or CAPD.

Nondiabetic Patients (NDM)

	**All NDM**			**HD**			**CAPD**		
	**ESRD-T0**	**ESRD-T6**	**p**	**ESRD-T0**	**ESRD-T6**	**p**	**ESRD-T0**	**ESRD-T6**	**p**
CD28null cells	162.5 (12.6–1209)	155.7 (11.8–1630)	NS	162.5 (12.6–1209)	170.9 (20–1630)	NS	190 (38–492)	129 (11–522)	NS
CD28null (%)	14.6 (1–50)	9.8 (0.5–53)	NS	14.6 (1–50)	12 (1.3–53)	NS	15.8 (3–34.6)	8.5 (0.5–37)	NS
CD4+CD28null	20.9 (0–550)	13.4 (0–810)	NS	32 (0–550)	26.7 (0–810)	NS	12.4 (1.9–164)	10 (0–156)	NS
CD4+CD28null (%)	4.1 (0–42.3)	2.1 (0–44.8)	NS	7.2 (0–42)	4.5 (0–44)	NS	2.5 (0.3–26)	1.3 (0–29)	0.04
CD8+CD28null	114 (12–735)	133 (11–820)	NS	116 (12–735)	145 (20–820)	NS	114 (36–407)	117 (11–366)	NS
CD8+CD28null (%)	43 (5.7–85)	41 (1.8–80)	NS	45.4 (8.6–77)	48.8 (11–79)	NS	43.4 (5.7–85)	39 (1.8–80)	0.04
Natural Killer cells	174.6 (0–617)	209 (79–550)	NS	175 (66–617)	238 (85–550)	NS	118 (0–310)	164 (79–308)	NS
Natural Killer cells (%)	14.6 (0–32.5)	14.4 (3.7–30.6)	NS	17 (4.5–32.5)	15 (3–30.6)	NS	13 (0–28)	10 (4.4–25)	NS
Treg cells	34.7 (9.9–130)	40.6 (6.7–110)	NS	48.8 (9.9–130)	40.8 (6.7–89)	NS	26.7 (16–107)	38.8 (12–110)	0.05
Treg cells (%)	2.8 (0.62–8.5)	2.5 (0.4–4.6)	NS	3.2 (0.6–8.5)	2.5 (0.4–4.6)	NS	2.6 (1.3–6.7)	3.1 (1–4.2)	NS
IRP	2.6 ± 0.6	2.8 ± 0.7	NS	2.5 ± 0.6	2.8 ± 0.7	0.05	2.7 ± 0.6	2.7 ± 0.9	NS

Diabetic Patients (DM)

	**All DM**			**HD**			**CAPD**		
	**ESRD-T0**	**ESRD-T6**	**p**	**ESRD-T0**	**ESRD-T6**	**p**	**ESRD-T0**	**ESRD-T6**	**p**
CD28null cells	206.8 (61–500)	257 (98–1396)	0.05	180 (61–500)	299 (98–1396)	0.01	357 (136–477)	225 (163–601)	NS
CD28null (%)	21.3 (4.4–34)	19.4 (6–41)	NS	21.7 (4.4–33.8)	22.7 (6.3–40.7)	NS	19.3 (15–23)	17 (8.9–24)	NS
CD4+CD28null	34.7 (14–279)	52.7 (15–203)	NS	30 (14–100)	52.7 (15–203)	0.02	61.5 (26–279)	52.5 (17–175)	NS
CD4+CD28null (%)	11.5 (1.5–24)	7.9 (1.3–19)	NS	12.5 (1.5–24)	11.5 (1.8–19)	NS	9.8 (2.9–19.8)	6.9 (1.3–+18)	0.05
CD8+CD28null	132 (47–400)	158 (25–1192)	0.05	130 (47–400)	212 (25–1192)	0.02	209 (102–378)	141 (103–540)	NS

CD8+CD28null (%)	61.3 (24–76)	63.8 (13–71)	NS	62 (24–76)	63 (13–71)	NS	59 (55–62)	63 (48–69)	NS
Natural Killer cells	141 (56–460)	246 (70–748)	0.02	137 (56–275)	266 (103–456)	0.01	250 (130–460)	198 (70–748)	NS
Natural Killer cells (%)	15.3 (7.7–31)	16.8 (3.9–35)	NS	14.7 (7.7–30.8)	18 (7.5–35)	NS	16 (9.7–23)	15.3 (3.9–30)	NS
Treg cells	33.7 (12–102)	34.7 (20–90)	NS	33.7 (12–54)	29.8 (20–80)	NS	39.7 (17–102)	38.3 (30–90)	NS
Treg cells (%)	3.1 (1.1–6.7)	2.1 (1.4–6.9)	NS	3.1 (2–6.7)	2.1 (1.4–6.2)	NS	2.9 (1.1–5.1)	2.6 (1.7–5)	NS
IRP	2.8 ± 0.7	2.8 ± 0.8	NS	2.8 ± 0.7	2.8 ± 0.6	NS	2.7 ± 0.5	2.7 ± 1.2	NS
